# Temporal variation and grade categorization of methane emission from LNG fueling stations

**DOI:** 10.1038/s41598-022-23334-2

**Published:** 2022-11-01

**Authors:** Yifan Wang, Jianfeng Tang, Donglai Xie, Fei Li, Ming Xue, Bo Zhao, Xiao Yu, Xiaojin Wen

**Affiliations:** 1 College of Pipeline and Civil Engineering, China University of Petroleum (East China), Qingdao, China; 2 Environmental Defense Fund, Surrey, Canada; 3 State Key Laboratory of Petroleum Pollution Control, CNPC Research Institute of Safety and Environmental Technology, Beijing, China; 4 Qingdao ENN Energy Corporation Limited, Qingdao, China

**Keywords:** Climate sciences, Environmental sciences

## Abstract

Natural gas is increasingly seen as the fossil fuel of choice for China as it transitions to renewable sources. The significant development of China’s LNG vehicle application and fueling stations and the urgency of climate changes make it particularly important to quantify methane emission from LNG stations, where the data are extremely rare. We carried out a pilot study on direct measurement and quantitative analysis of methane emission from five LNG fueling stations located in Shandong, China following the standard stationary EPA OTM 33A method. The measured methane emission of these five stations vary from 0.01 to 8.76 kg/h. The loss rates vary from 0.004 to 0.257%. We demonstrated that the emission from LNG stations consist of continuous and intermittent contents. The intermittent emission shows a strong temporal variation. If a station is only monitored for 20 min, it may either under-estimate or over-estimate the total emission. Both the distribution of emission events and total emission rates among different stations are highly skewed. We found that these LNG fueling station emission can be categorized into 3 grades, as low, medium and high, corresponding to emission rates below 0.1 kg/h; between 0.1 and 1 kg/h and above 1 kg/h, which can be characterized by the measured average methane concentration enhancement.

## Introduction

With the growing pressure on providing enough energy for economy development while reducing Green House Gas (GHG) emission, natural gas is increasingly seen as the fossil fuel of choice for China as it transitions to renewable sources. In recent years, the natural gas consumption in China is showing a rapid growth trend, helping to improve regional air quality. During the “13th Five-Year Plan” period, the average annual growth of China's natural gas consumption exceeds 20 billion cubic meters, with an average annual growth rate of more than 10%. In 2019, China's natural gas consumption exceeded 300 billion cubic meters and reached 320 billion cubic meters in 2020, an increase of approximately 127 billion cubic meters over 2015^1^. In China’s latest “14th Five-Year Plan”, domestic natural gas production is expected to keep increasing^2^. Natural gas is gradually developing into one of China's main energy sources.

Among various natural gas applications, transportation is an important sector, where natural gas is supplied in the form of either Compressed Natural Gas (CNG) or Liquefied Natural Gas (LNG) to vehicles. In 2012, the National Development and Reform Commission issued the Nation’s “Natural Gas Utilization Policy”, encouraging the construction of natural gas fueling facilities for automobiles and ships. For the first time, natural gas vehicles, especially liquefied natural gas vehicles, were included in the priority development category^3^. In 2016, the National Energy Administration studied the revised policy on natural gas utilization and proposed to actively promote the efficient utilization of natural gas and accelerate the construction of fueling facilities and equipment^4^.

In China, there were about 6.7 million natural gas vehicles by the end of 2018, an increase of 620,000 over the past 10 years, of which approximately 440,000 were LNG vehicles^5^. As an integrated part of natural gas supply chain, LNG fueling stations have to grow rapidly to meet the fueling demand imposed by the increasing numbers of LNG vehicles. In 2011, there were only 100 LNG fueling stations^2^, while in 2018 there were about 3500 nationwide. It is predicted that, there will be 7500 LNG fueling stations in 2025^3^. Although fueling switching to natural gas could provide a climate benefit relative to gasoline or diesel fuel as natural gas has lower carbon per unit of energy than oil, the potential for emission of methane from natural gas value chain has been identified as a concern. Methane emission from the energy sector have become the second largest source of anthropogenic emission^6^.

Alvarez et al. demonstrated that when the GHG emission from light duty gasoline vehicle are 0.1056 kg CH_4_ and 86.2 kg CO_2_ per mmBtu HHV (Higher Heating Value), the maximum “well-to-wheels” natural gas leak rate needs to be below 1.6% to obtain an immediate climate benefit for the fuel to switch from gasoline to CNG. For heavy duty diesel vehicles, when their GHG emission are 605 mg CH_4_ and 90,000 mg CO_2_ per ton-mile, the maximum natural gas leak rate needs to be 1.0% for immediate climate benefit when switching to CNG^7^. Camuzeaux et al.^8^ further studied the influence of methane emission and vehicle efficiency on the climate implications of heavy-duty natural gas trucks. Using reference case assumptions reflecting then-currently available data, they found that converting heavy-duty truck fleets leads to damages to the climate for several decades. Their range of results indicates that these fuel switches have the potential to produce climate benefits on all time frames, but combinations of significant well-to-wheels methane emission reductions and natural gas vehicle efficiency improvements would be required^8^. In addition, Nam et al.^9^ pointed out that the CH_4_ emission of trucks are about 40% higher than that of cars. Currently LNG vehicles in China are mostly trucks, not cars.

It could be seen from above studies that climate benefit of natural gas application in transportation sector replacing gasoline or diesel really depends on the methane emission through the natural gas value chain. While there are plenty of measurements and data synthesis for the production, transmission, storage and distribution of natural gas^8^, as well as emission from natural gas vehicles, there are very few actual measured emission data on LNG fueling stations.

Clark et al.^10^ used hand-held methane detectors to identify methane emission sources at six LNG fueling stations, and used a full flow sampling system to quantify the emission rate. Their detected continuous leaks at six LNG stations ranged from 0.01 to 53.1 g/h with continuous leak rates at three stations below 1 g/h (average 12.8 g/h; MU <  ± 0.6 g/h).

In summary, the significant development of China’s gas fueling stations and natural gas vehicles and the urgency of climate changes make it particularly important to quantify methane emission from LNG stations, where the data are extremely rare now. In view of the above backgrounds, we carried out a pilot study on direct measurement and quantitative analysis of methane emission from five LNG fueling stations located in the east of China, with the aim of accelerating the promotion of methane emission measurement and obtaining empirical data to build China’s LNG fueling station emission factors.

## Instrument and emission quantification

### LNG fueling stations

The footprint of a typical LNG station usually consists of vehicle fueling region, LNG offload region and LNG storage region. As summarized by Clark et al.^10^ methane emission from LNG fueling stations can occur at any process of the station itself (plumbing leaks, station tank venting), the vehicles being fueled (fuel system leak and venting; uncombusted exhaust), as well as the LNG delivery tanker (pipeline leak and tank venting).

Five LNG fueling stations were selected for the emission measurement. Their capacity and configurations were listed in Table 1. The following factors were considered when selecting these stations for effective application of OTM 33A method in the EPA official website, which are used as the main basis for the selection of LNG fueling stations^11,12^.

1. An accessible and safe downwind site for instrument installation;
2. A relatively consistent wind condition blowing from the source to the point of measurement;
3. Zero or few obstructions between the source and the measurement point;
4. No other nearby emission sources.

**Table 1:** Main parameters of 5 gas fueling stations.

Station number	Storage capacity	Daily unloading	Unloading frequency	Number of gas dispenser	Vehicle type	Note
1^#^	One 60 m^3^ tank	20 T	Once a day	2	Heavy truck Cement mixer	Built in recent years
2^#^	One 60 m^3^ tank	10–15 T	Every 1–2 days	1	Heavy truck Bus	Built in conjunction with a gas station; Surrounding is under construction with small traffic flow
3^#^	One 60 m^3^ tank	20 T	Once or twice a day	2	Heavy truck Bus	BOG connected to distribution pipe network; Open area around the station
4^#^	Two 60 m^3^ tanks	40 T	2–3 times a day	4	Heavy truck Bus	BOG connected to city pipe network; High traffic density
5^#^	One 60 m^3^ tank	20 T	Once a day	2	Oil tank truck Heavy truck	Surrounding flat and open, no shelter

Operators of these stations were contacted 1–2 days before the measurement for our site access, but no evidence showed that they have performed any extra maintenance work to fix leaks before or during measurement.

### Instruments measurement procedure

The methane emission quantification methods of the oil and gas industry mainly include top-down and bottom-up methods^13^. The combination of bottom-up and top-down approaches can improve the quality of the measured emission data^14–16^. In this research the actual methane emission from LNG fueling stations were measured and quantitatively analyzed by the top-down measurement.

In the measurement, we continuously monitored methane concentration downwind of LNG fueling stations at specific sites using high precision methane concentration analyzers (Picarro G2301 and G4301), while wind direction and speed were measured simultaneously with Gill Wind master precision three-dimensional ultrasonic anemometer.

**Figure 1:**
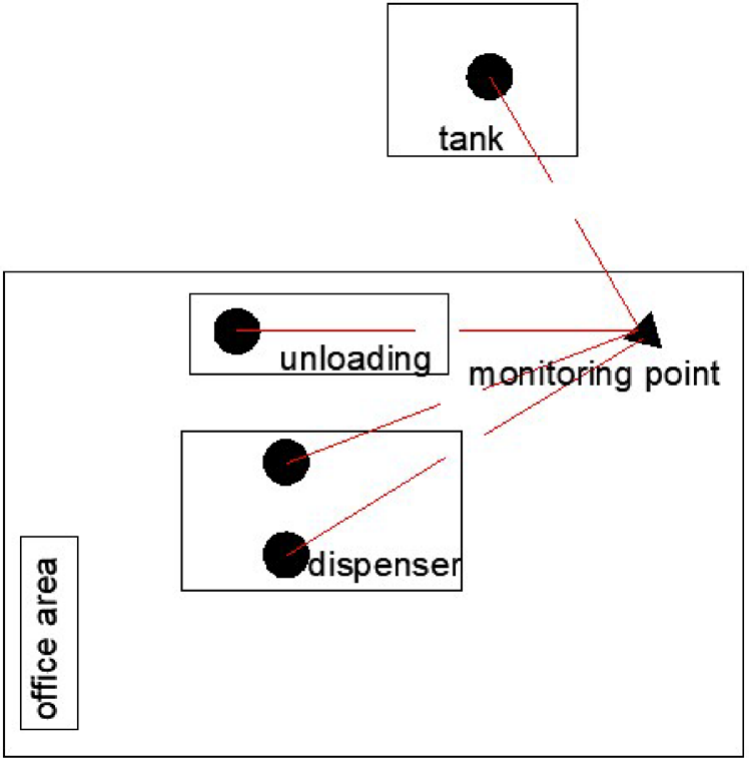
Sketch of a typical LNG fueling station footprint and the location of the monitoring point.

The specific measurement steps are as follows: according to the real-time prevailing wind direction of the day in the station, Picarro instrument was set up at a feasible measurement position under the wind direction of the fueling station while avoiding the entrance and exit of the LNG fueling station and narrow intersections; Install the three-dimensional anemometer and the overall wind speed and direction of the gas fueling station and other meteorological conditions could be obtained. Meanwhile, observe the relevant methane concentration fluctuation image on the methane concentration analyzer and output real-time concentration data. Figure 1 shows the approximate footprint of an LNG fueling station and the location of potential emission sources and monitoring point.

A total of 5 stations were measured for methane concentration in the downwind direction for 9 days in different time periods during the measurement. Some detailed information is listed in Table 2.

**Table 2:** Some detailed information of measurements.

Station	Date	Measuring period	Picarro model	Height of sampling port (m)	Prevailing wind direction	Dominant wind speed (m/s)
1^#^	2020.9.16	15:30–21:10	G2301	2.2	Southeast	0.64
1^#^	2020.9.17	10:30–16:50	G2301	2.2	Southeast	0.74
1^#^	2020.10.21	13:40–16:50	G4301	2.2	Southeast	0.67
1^#^	2020.10.22	9:00–14:40	G4301	2.2	Southeast	0.77
2^#^	2020.10.23	9:30–16:00	G4301	2.2	Southwest	3.17
3^#^	2020.10.24	10:26–16:24	G4301	2.2	Southeast	1.33
4^#^	2020.10.25	9:18–11:57	G4301	2.2	Southeast	1.51
4^#^	2020.10.26	8:55–11:35	G4301	2.2	Southwest	0.53
5^#^	2020.10.27	12:51–13:51	G4301	1.7	Southwest	2.18

Picarro analyzer is located 23 m downwind of the nearest dispenser.

During the measurement, the number of vehicles being fueled, as well as the time when a delivery tank offload LNG to the station were recorded. However, when we analyzed the emission data, no correlation between the emission and these events could be found. Hence these activity data are not reported here.

### Emission quantification

The methane emission rates were quantified following the standard stationary EPA OTM 33A method^11,17,18^ by the Matlab-based point source Gaussian analysis program. Details of the method can be found elsewhere. Brantley et al. used the OTM 33A method to measure the short-term emission rates of 210 oil and gas production platforms in and around Texas^16^. Robertson et al. used this method to measure methane emission fluxes in four basins in the United States^19^.

The standard stationary OTM 33A method usually requires approximately 20 min measurement to quantify an emission rate from an upwind target source. As we monitored hours of methane concentrations in the measurement, a pseudo- instantaneous emission rate can be obtained by quantifying emission rates at every 20 min interval, and the temporal methane emission variation of these LNG fueling stations can be revealed.

Following the standard practice of OTM 33A^11,12^, the raw methane concentration data and meteorological data require the implementation of certain quality control criteria before performing the next step of the calculation. These criteria mainly include wind speed greater than or equal to 1 m/s, turbulence intensity less than or equal to 0.22, atmospheric stability greater than 1. Any data that do not meet these standards were excluded from the calculation.

## Results and discussion

### Downwind methane concentration

**Figure 2:**
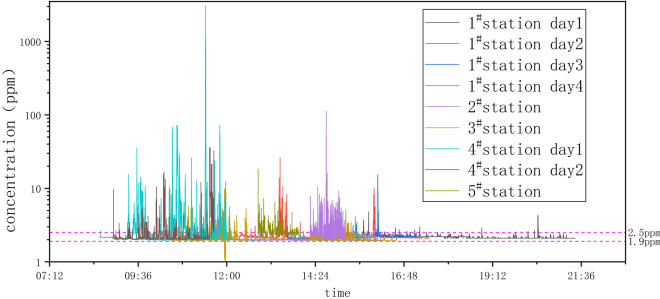
Measured methane concentrations downwind of LNG fueling stations (measurement conditions are listed in Table 2).

A total of 9 continuous measurements of methane concentration at a fixed point in the downwind direction were carried out at 5 LNG fueling stations. Each continuous measurement time ranged from 2 to 7 h. The measured methane concentration is shown in Fig. 2. The methane concentration of each station varies over time, and the measured baseline methane concentration is between 1.9 and 2.5 ppm. The maximum concentrations of the five stations vary significantly. Because the distance between the measurement instrument and the station is roughly the same, the emission rate from each station will be very different.

**Figure 3:**
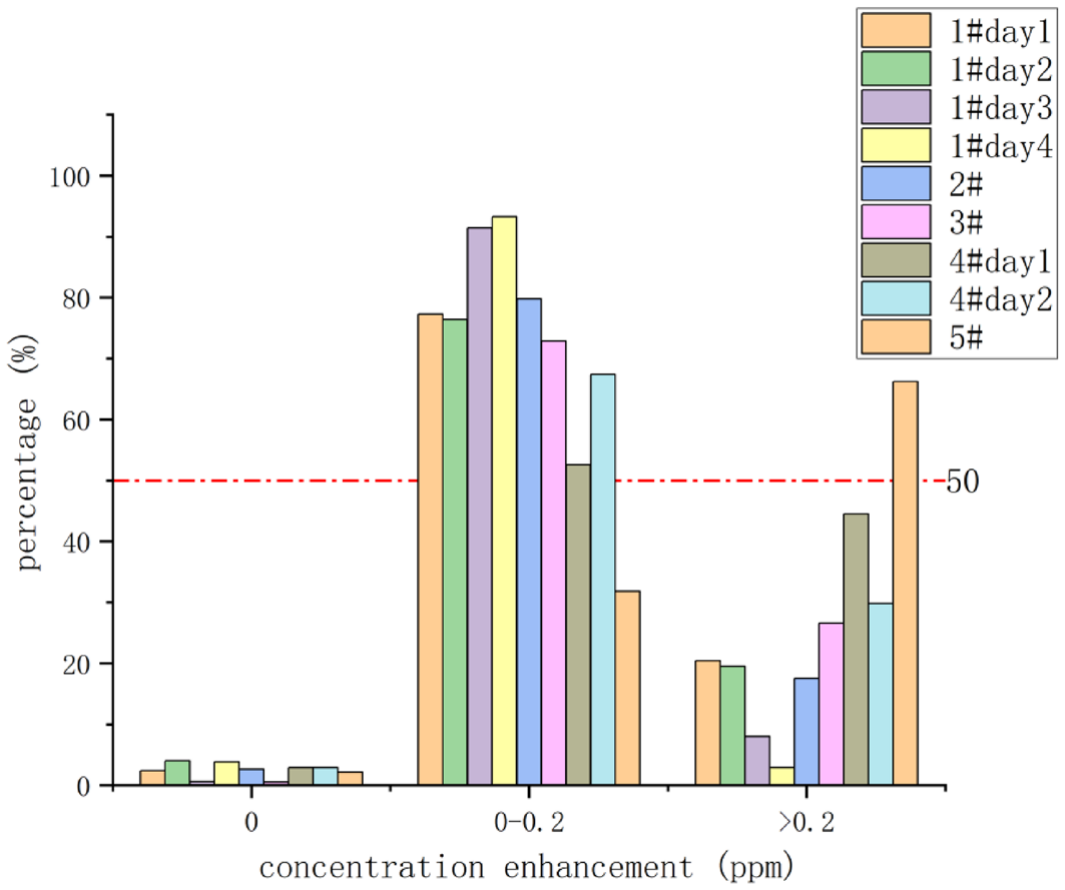
Statistical distribution of measured methane concentration enhancement.

Figure 3 shows the statistical distribution of measured gas concentration enhancement. We divided methane concentration enhancement into three categories: 0; 0–0.2 ppm; > 0.2 ppm. It can be seen that the majority (> 50%) of the measured methane concentration enhancement fall into the 0–0.2 ppm category. The > 0.2 ppm account for about 20% of the total number in most stations, while the amount of data with zero methane concentration enhancement account for the least proportion, which is lower than 5%.

### Methane emission rate

**Figure 4:**
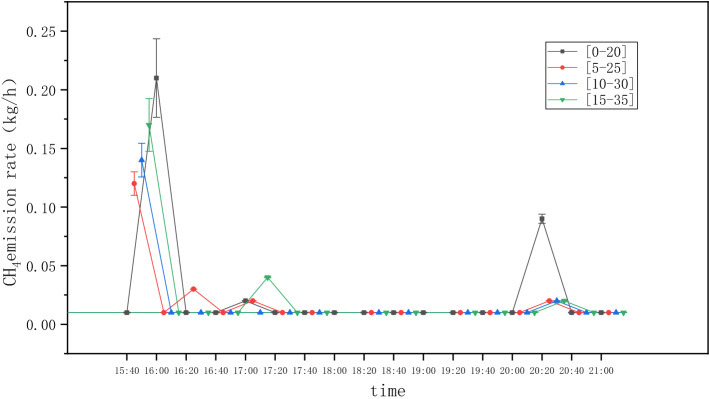
Influence of starting point on the quantified 20 min average emission rate (1^#^ Station on Sept 16, 2020).

The Gaussian point source method, OTM33A issued by the U.S. Environmental Protection Agency, is used to calculate the methane emission rate. Usually 20 min concentration measurement is required to acquire an emission rate estimation^12^. In our study, the measurement time is between 2 and 7 h. Hence in our analysis, we break down the whole measuring period to several sequential 20 min sections, and data within each section is analyzed to obtain an average emission rate within that section. A series of pseudo-instantaneous emission rates can then be derived to reveal the temporal emission variation for the total measured time periods. The starting point of the first 20 min section may have some influences on each averaged emission rate. With the data obtained for Station #1 on Sept 16, 2020 as an example, Fig. 4 shows that when we selected the starting points for the first 20 min section as 0, 5, 10 and 15 min after the kick-off of the concentration measurement, the averaged emission rates profile change slightly. When we calculated the total average emission rate over the 5 h measuring period, the numbers we obtained are 0.0271, 0.0200, 0.0193, 0.0233 kg/h, respectively, for these 4 sets of processing. It indicates that the selection of time interval for quantifying emission rate does affect the total average emission rate, but within a limited range.

One can imagine that there are two sources contributing to the emission: continuous emission like pipeline or equipment leakage, and intermittent emission caused by operations like LNG fueling, unloading or even Boil Off Gas (BOG) venting. In this study, we define the continuous emission as the one takes place at the time periods when the measured methane concentration is less than 2.5 ppm, while the remaining is defined as intermittent emission.

For the 5 stations measured, Table 3 shows the total averaged emission rates, and their contribution from continuous and intermittent emission, as well as the loss rate, where it is defined as

1 $${\text{Loss}}\,{\text{ rate}} = {\text{CH}}_{{4}} {\text{emission}}\,{\text{ rate}} \times {24}/({\text{Daily}}\,{\text{ LNG}}\,{\text{ sales}})$$

**Table 3:** Average methane emission from LNG fueling stations.

LNG station	Date of measurement	Emission rate (including Standard Deviation) (kg/h)	Average continuous emission (%)	Average intermittent emission (%)	Daily LNG sales (kg)	Loss rate (kg/h)
1	Sep. 16	0.03 (0.05)	19	81	2.3 × 10^4^	0.004%
1	Sep. 17	0.08 (0.19)	19	81	2.3 × 10^4^	0.004%
1	Oct. 21	0.02 (0.04)	19	81	2.3 × 10^4^	0.004%
1	Oct. 22	0.01 (0.03)	19	81	2.3 × 10^4^	0.004%
2	Oct. 23	0.38 (0.75)	27	73	1.2 × 10^4^	0.076%
3	Oct. 24	0.11 (0.10)	37	63	2.6 × 10^4^	0.010%
4	Oct. 25	8.76 (20.59)	9	91	4.2 × 10^4^	0.257%
4	Oct. 26	0.24 (0.35)	9	91	4.2 × 10^4^	0.257%
5	Oct. 27	0.30 (0.13)	44	55	2.1 × 10^4^	0.034%

Daily loss rate was calculated as the ratio of the daily emission volume to the daily LNG sales.

It can be seen from Table 3 that the standard deviation of emission rates for each station is quite high comparing to the estimated average value, which is an indication that the emission rates for each 20 min quantification are highly scattered. This is consistent with the emission pattern shown in Fig. 4: while for the majority of the time the emission is very low, there are intermittent emission peaks.

Another source of the high standard deviations may come from the uncertainties associated with the measurement. OTM 33A itself has its own uncertainties. OTM 33A inherent uncertainty due to the assumptions of the computational model is affected by the length of various time periods, data rates, and wind filters^12,20,21^. In addition, there are uncertainties in the measurement process, where the major factors will be the downwind distance and height differences between the emission source, or possible multi-sources, and the measuring point. Experiments by Rachel et al.^17^ showed that the source height has little effect on the OTM 33A method. However, the existence of potential emission sources may cause a certain deviation in the downwind distance. The main emission sources of conventional LNG fueling stations come from the fueling area, storage tank area and unloading area, among which the fueling area with two dispensers is about 18 m × 20 m; fueling area with 4 dispensers is about 40 m × 20 m; unloading area is 20 m × 6 m; and the storage tank area is 15 m × 20 m. We made the extreme case assumption for a measured station that when the main potential emission sources exist, the downwind distance is determined to be 28 m by the average of the distance and sum between the measurement point and each emission source, which is 5 m different from the downwind distance used in our original calculation. In this case, the emission flux changes by 37.9%. Given that there were sixteen 20 min measurement events at our measurement sites, the increased uncertainty in the average emission rate during each 20 min period was approximately 9.475%, which means that repeated measurements could reduce the uncertainty of randomly distributed measurements.

The methane emission rates of the 5 stations can be seen varying significantly, in the order of magnitude, the average methane emission rate of 1^#^ gas fueling station is the smallest, in the magnitude of 10^–2^ kg/h, while emission from stations of 2^#^, 3^#^, 5^#^ are in the order of 10^–1^ kg/h, and emission rate of 4^#^ gas fueling station is the highest, reaching 4.5 kg/h. From the perspective of the contribution ratio, it can be seen that the intermittent emission of gas fueling stations are the main source of emission. The loss rates of the 5 fueling stations also vary significantly. The smallest loss rate is only 0.004% for 1^#^ LNG fueling station, and the highest loss rate comes from 4^#^ fueling station, which is 0.257%.

**Figure 5:**
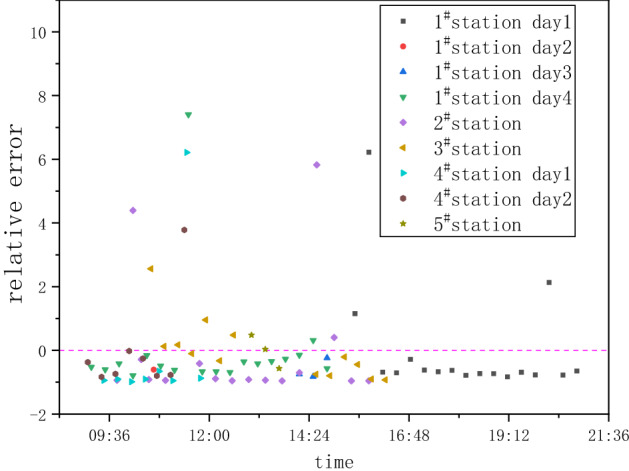
Relative error of different measurement periods.

As have discussed previously, the methane emission from LNG fueling stations show strong temporal dependence. Hence it will introduce significant bias if one takes a short period time of measurement and try to use that result to represent the overall averaged emission. Based on the data collected by this study, the error introduced by any 20 min measurement is estimated, comparing the 20 min measurement value to the averaged value over the whole period of 2–7 h, and the result is illustrated in Fig. 5. Most of the relative errors are less than zero and are concentrated between − 1 and 0, which may make the short-period measurement method average lower than the long-period measurement average, resulting in an underestimation of methane emission. Even in some time periods, the error may reach 10, resulting in a serious overestimation of methane emission from gas fueling stations. Therefore, a comprehensive estimation of methane emission from gas fueling stations may require a longer continuous measurement period.

**Figure 6:**
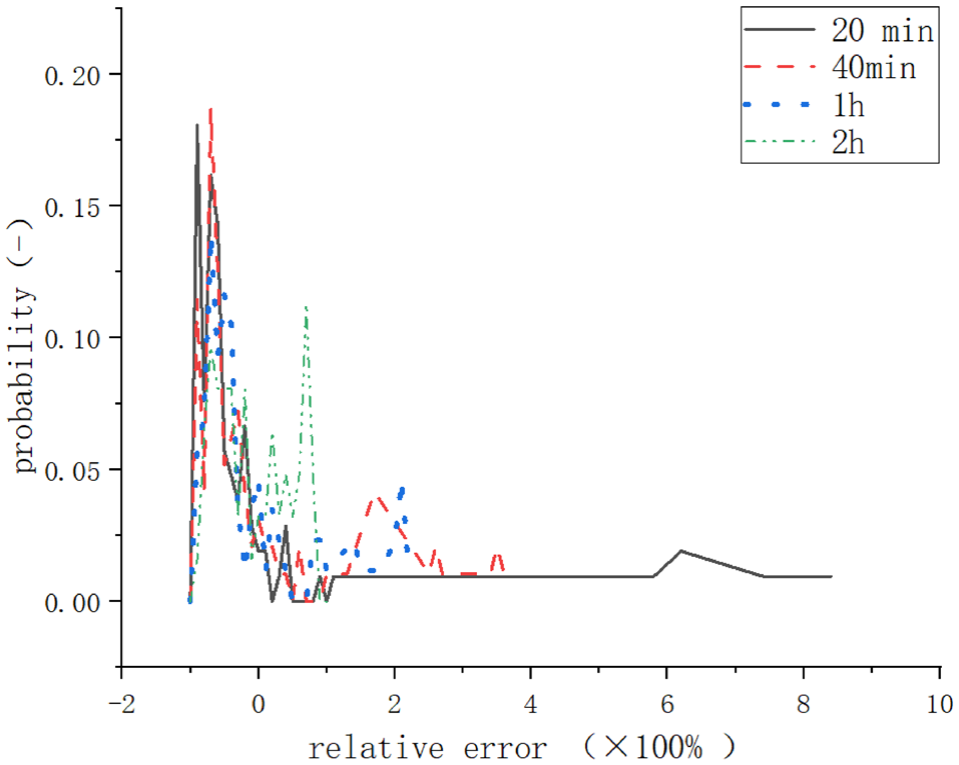
Relative error of different data analysis period.

Figure 6 shows the influence of different measuring time lengths on the relative error probability. When measuring time length of 40 min, 60 min, 1 h and 6 h apply, we assume the measured emission rates are the average values from the continuous 20 min measurements within that time range. As the measurement time length increases, the probability of under-estimating decreases. Actually, when the measuring time of 2 h is used, it looks like the probabilities for under-estimating and over-estimating balance each other.

### Emission event distribution

**Figure 7:**
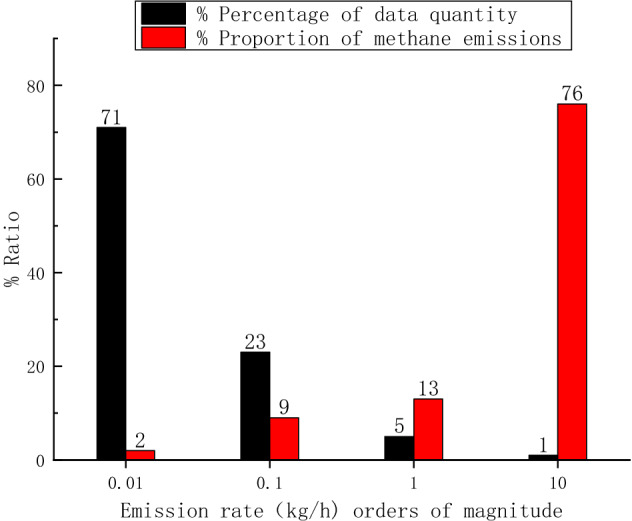
Population distribution of emission events and total emission as function of emission size.

If the emission in each 20 min is regarded as an individual event, the population distribution of these emission events and total emission as a function of emission size are shown in Fig. 7, it can be seen that 71% of the emission events are at the level of 0.01 kg/h, but it contributes to only 2% of the total emission, while 76% of the methane emission come from 1% of the events with emission rate at the level of 10 kg/h. This is a typical fat tail distribution.

In addition, there is also a fat tail distribution of methane emission rates between gas fueling stations. It can be seen from Table 3 that among the 5 stations, 4^#^ gas station has an excessively large emission rate of 4.5 kg/h, more than five times of the total emission rate of the other four stations, i.e., a small fraction poorly operated fueling stations contribute to a large fraction of total emission. This result is consistent with the general emission distribution in natural gas infrastructure, as has been described by Zavala-Araiza et al.^22,23^.

### Emission grade categorization

The application of Matlab-based EPA OTM 33A point source Gaussian emission quantification requires skilled scientists or engineers to conduct tedious computation efforts, which may not be feasible for routine industry emission measurement and monitoring. In the real world, if it is possible, it may be beneficial to categorize an emission grade for a certain station by the measured downwind methane concentration, without calculating an emission rate number. By observing the emission rates that have been measured for the 5 LNG stations, referring to classification scheme for urban natural gas leakage of Weller et al.^24^, we decide to classify the methane emission rates from LNG stations into three levels: low, medium and high, according to the order of magnitude of emission rate, as shown in Table 4.

**Table 4:** LNG station emission grade categorization.

Emission categorization	Emission rate range (kg/h)	Average methane concentration enhancement (ppm)
Low	< 0.1	< 0.22
Medium	0.1–1	0.22–0.94
High	> 1	> 0.94

**Figure 8:**
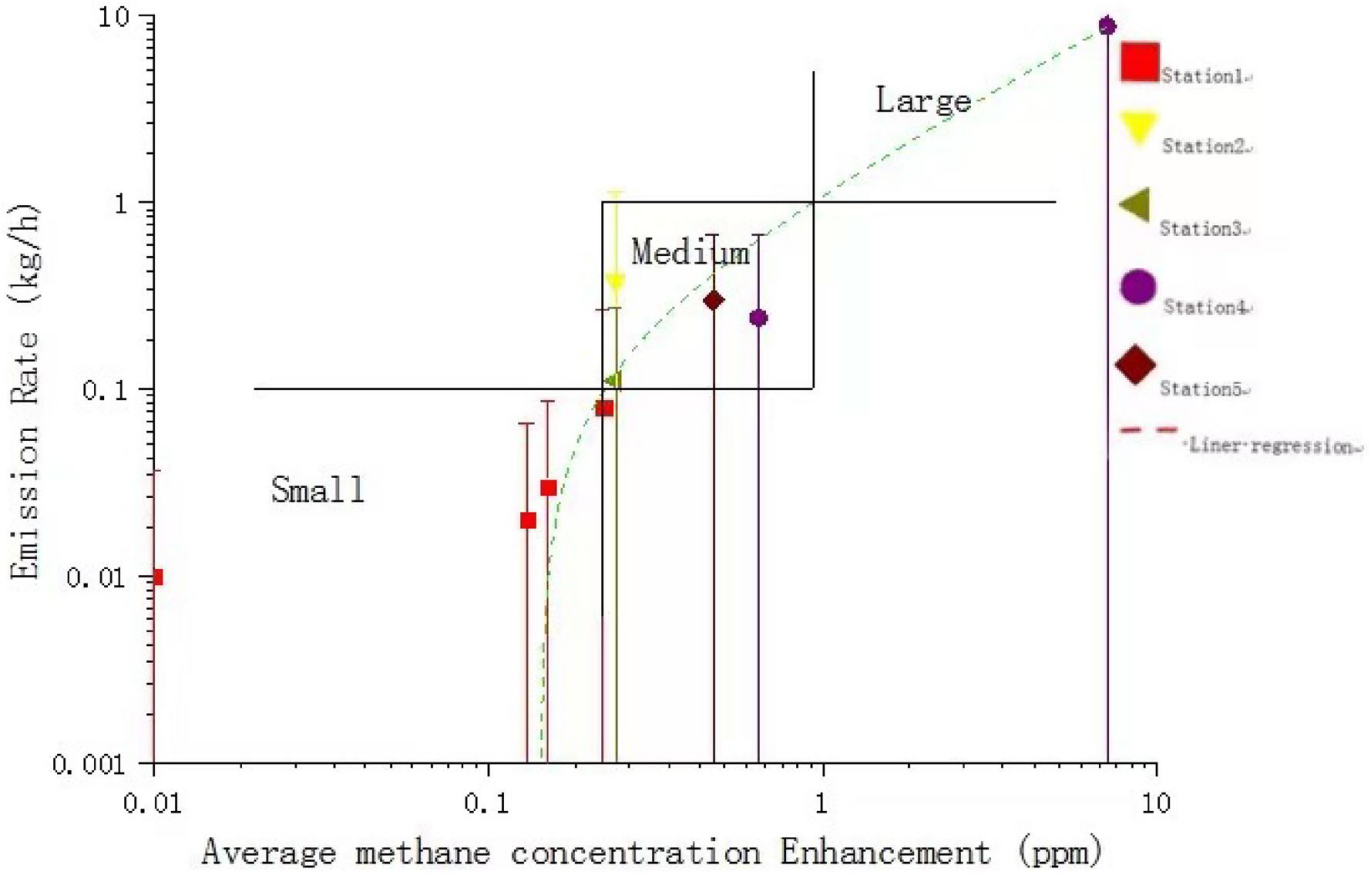
Correlation between average methane concentration enhancement and the methane emission rate, and emission grade categorization.

By analyzing the measured methane concentrations approximately 23 m downwind of the nearest dispenser, we found that there is a certain correlation between the measured average methane concentration enhancement and the methane emission rate, as shown in Fig. 8. We define the average methane concentration enhancement (***C***_ae_) as the measured average methane concentration (***C***_av_) minus the methane background concentration (***C***_bg_):

2 $${\varvec{C}}_{{{\text{ae}}}} = {\varvec{C}}_{{{\text{av}}}} - {\varvec{C}}_{{{\text{bg}}}}$$

It can be seen from Fig. 8 that the emission rate increases almost proportionally with the measured average methane concentration enhancement, with the dotted line a liner regression of the emission rate and methane enhancement. The concentration enhancements that demarcate low/medium and medium/high emission are approximately 0.22 ppm and 0.94 ppm, respectively. However, it should be noted that:

1. The current methane concentration was measured approximately 23 m downwind of the nearest dispenser. If the measurement location changes, the current assessment criteria should vary as well.
2. In the current study, only one station falls into the large emission category. More measurements are needed to improve the current categorization assessment.

## Key conclusions and recommendations

Through the analysis of the above results, the following conclusions can be clearly drawn.

Among these five LNG stations, the measured methane emission rates vary from 0.01 to 8.76 kg/h. The loss rates vary from 0.004 to 0.257%. The emission from a station consist of continuous and intermittent contents. The intermittent emission shows a strong temporal variation. If only the traditional 20 min measurement time is used, it may either under-estimate or over-estimate the total emission.

Both the distribution of emission events, and the distribution of emission rates among different stations show strong “fat tail” distribution. For emission events, 76% of the methane emission come from 1% of the events with emission rate at the level of 10 kg/h. Among the 5 stations, 4^#^ gas station has an excessively large emission rate of 4.5 kg/h, more than five times of the total emission rate of the other four stations.

We categorize LNG fueling station emission into 3 grades, as low, medium and high, corresponding to emission rates below 0.1 kg/h; between 0.1 and 1 kg/h and above 1 kg/h. 10^–2^, 10^–1^, and 10^0^ kg/h respectively. The average methane concentration enhancement can be used to identify the emission grade.

While the current study shows strong temporal variation of methane emission from LNG fueling stations, it should also be noted that oil and gas methane emission also varies spatially^25^. The measurement data of these five LNG fueling stations in East China region cannot represent their emission in China, where meteorological conditions and operators change from province to province, from city to city. More measurements are needed at LNG stations across the country so that the average emission estimate is representative and can be included in a national emission inventory if created.

## Data Availability

The datasets generated during and/or analyzed during the current study are available from the corresponding author on reasonable request.
